# Association Between Mandibular Third Molar Position and Recurrent Pericoronitis: Protocol for a Cross-Sectional Study

**DOI:** 10.2196/72682

**Published:** 2026-01-21

**Authors:** Suwarna Dangore- Khasbage, Tanaya Teredesai

**Affiliations:** 1 Department of Oral Medicine and Radiology Sharad Pawar Dental College and Hospital Datta Meghe Institute of Higher Education and Research Wardha, Maharashtra India

**Keywords:** eruption, molar, orthopantomogram, radiograph, wisdom teeth

## Abstract

**Background:**

Pericoronitis is a common pathological condition associated with mandibular third molars that may cause pain and discomfort. This condition may be chronic, exhibiting episodic symptoms that last for a few days to weeks and recurring multiple times in less than a year. The operculum covering the erupting mandibular third molars may become obscured by the eruption of maxillary third molars. Such recurrent traumas might exacerbate the symptoms and lead to ulcerations. With clinical monitoring at regular intervals and with the help of radiographic examinations, clinicians can develop the most effective treatment plan.

**Objective:**

This study aims to determine the association between mandibular third molar position and recurrent pericoronitis.

**Methods:**

This cross-sectional study will include 200 patients having partially impacted mandibular third molar with recurrent pericoronitis. Patients aged 18-40 years with occurrence of pericoronitis will be included in this study. The impacted tooth’s side and the symptoms associated with pericoronitis will be recorded during clinical examination. All these patients will be evaluated using panoramic radiographs to assess the position of the unerupted/impacted mandibular third molar.

**Results:**

The duration of this study will be 6 months from October 2025 to April 2026. Approval for this study has been granted by the institutional ethics committee of Datta Meghe Institute of Higher Education and Research (deemed to be university), Sawangi, Wardha (DMIHER (DU)/IEC/2024/53). In panoramic radiographs, the impaction status of the mandibular third molar will be evaluated based on Winter’s and Pell and Gregory classification systems. Patients’ data will be recorded and analyzed for statistical significance.

**Conclusions:**

The detection of the position and intervention at the early stage for pericoronitis in individuals with impacted third molars is vital.

**International Registered Report Identifier (IRRID):**

PRR1-10.2196/72682

## Introduction

Pericoronitis is a painful inflammatory condition of the gingival tissue surrounding the crown of a partially erupted tooth. It is often related with the positional relationship of the third molar with the surrounding hard and soft tissues. The condition is more common with mandibular third molars as compared to maxillary third molars [1,2].

The partially erupted tooth is nestled in the posterior recesses of the mandibular arches under the soft tissue or inside the osseous bounds and adopts a mesioangular, horizontal, or distoangular inclination. This abnormal position of the tooth provides an isolated plaque-retentive situation. Further, this improper placement of the partially erupted third molar creates an environment that is conducive to recurrent episodes of infection, swelling, and discomfort because of the trapping of food particles and harmful bacteria beneath the gingival operculum. This leads to the inflammation of the surrounding soft tissue, that is, pericoronitis. Nevertheless, this condition becomes recurrent due to anatomic inaccessibility for dental hygiene procedures, the pressure from the neighboring teeth, and the trauma from erupting maxillary third molars [1,3].

The common symptoms of pericoronitis include fever, soreness, swelling, pus discharge, and trouble in opening the mouth. This condition may be chronic, exhibiting episodic symptoms that last for a few days to weeks and recurring multiple times in less than a year [4]. If it is not treated in time, it can spread to the surrounding soft as well as hard tissues leading to complications such as maxillofacial space infection, buccal fistula, and osteomyelitis of the jaw [5]. Thus, considering the number of symptoms and accompanying complications, it is recommended to evaluate the position of an impacted third molar that is more at risk to be affected by pericoronitis so as to initiate early intervention.

Few previous studies [1,2,6,7] have reported associations between the position of the mandibular third molar and pericoronitis. However, the results are quite contradictory. Furthermore, despite numerous studies exploring the etiology and management of pericoronitis, a significant research gap remains in understanding the precise correlation between the positional relationship of third molars and the frequency or severity of recurrent pericoronitis. This gap contemplates the need for undertaking this study.

The aim of this study was to determine the mandibular third molar position and its association with recurrent pericoronitis. The research question is as follows: what is the association between the mandibular third molar position and recurrent pericoronitis?

## Methods

### Ethical Considerations

This cross-sectional study will be performed in the Oral Medicine and Radiology Department, Wardha, Maharashtra, India. The duration of this study will be 6 months from October 2025 to April 2026. Approval for this study has been granted by the Institutional Ethics Committee of Datta Meghe Institute of Higher Education and Research (deemed to be university), Sawangi, Wardha (DMIHER (DU)/IEC/2024/53). The data collected will not include any identifiable information. The objectives of the study will be thoroughly explained to each patient, and informed written consent stating data confidentiality, right to withdraw, and compensation will be obtained prior to any examination.

### Study Population

This study recruits patients visiting the outdoor patient department of Oral Medicine and Radiology with a history of pain, hyperemia, edema associated with the mandibular third molar of at least two episodes within a duration of 12 months, and affecting the same partially erupted or impacted tooth.

The inclusion criteria for the study will be patients aged 18-40 years, patients with history of pain, patients with recurrent pericoronitis treated by conservative methods, and patients with teeth in the vicinity of pericoronitis that are healthy and whole. The exclusion criteria will be history of extraction of third molar, missing third molar, fracture due to trauma to impacted or partially erupted third molar, and pregnant or lactating females. The sample size for this study will be determined as shown in Textbox 1 [8,9].

**Sample size estimation.**

**Sample size**

The sample size is calculated based on Daniel formula [9] for sample size estimation:

n = (Z² * P * (1 - P)) / d²

Where:

- Z represents confidence level. Confidence level is set at 95%; then the value of Z = 1.96.
- P is the anticipated proportion or prevalence. A common assumption for P is 0.5.
- d is the margin of error or precision. A value of 0.05 is often used to ensure high precision and minimize the error in the estimate.

Z = 1.96

P = prevalence of mandibular third molar with pericoronitis = 54.1% = 0.541

E = acceptable margin of error, which is 7% and equivalent to 0.07

n = 1.96 2 * 0.541 * (1-0.541) 0.072 = 194.7 = 200 patients needed in the study [8]

To ensure adequate power and to account for potential dropouts or loss to follow-up (≈5%) during the 12-month follow-up period, the final sample size was rounded up to 200 participants.

### Methodology

A total of 200 patients with recurrent pericoronitis and willing to participate will be selected for this study over a period of 1 year. These patients will be chosen among those reporting to the outpatient department of the Oral Medicine and Radiology department. Recurrent pericoronitis will be typically diagnosed when 2 or more distinct episodes of pericoronitis occur within a duration of 12 months, affecting the same partially erupted or impacted mandibular third molar. After obtaining the informed written consent, clinical as well as radiological evaluation of each patient will be performed.

The first step will be clinical evaluation, which includes a detailed collection of each patient’s medical and dental history, information about the duration of the painful episodes, and frequency and severity of painful episodes associated with an impacted mandibular third molar. Clinical examination using diagnostic instruments will focus on identifying the symptoms of pericoronitis, assessing the periodontal and gingival health of the affected third molar with a Williams’s periodontal probe, and determining the carious status of adjacent teeth.

The second step will be to analyze the position of the third molar by using panoramic radiography. To evaluate its angulation and position, Winter’s and Pell and Gregory classification systems will be applied to categorize partially erupted or impacted third molars [10-12]. These diagnostic measures aim to provide a comprehensive understanding of the clinical and radiographic characteristics of pericoronitis cases, ensuring accurate data for subsequent analysis and interpretation.

The radiographs will be assessed by a postgraduate student who will be blinded and a senior experienced radiologist who will not be blinded. Thus, this study will be single blinded. Interobserver reliability will be calculated by intraclass correlation before the inclusion of the radiographs. Patients in the age group of 18-40 years will be thoroughly examined to ensure comprehensive data collection. All the participants will be followed for future recurrent episodes for a duration of 12 months with an interval of every 3 months or upon patient-reported symptoms.

## Results

The duration of this study will be 6 months from October 2025 to April 2026. Approval for this study has been granted by the Institutional Ethics Committee of Datta Meghe Institute of Higher Education and Research (deemed to be university), Sawangi, Wardha (DMIHER (DU)/IEC/2024/53). Only those individuals who meet the inclusion and exclusion criteria will be considered as study participants. The parameters that will be determined are mandibular third molar position and episodes of recurrent pericoronitis. Data analysis will evaluate the primary outcome that includes the association between mandibular third molar position and recurrence of pericoronitis as well as secondary outcomes such as number and severity of episodes, angulation and depth of impaction, and comparison between Winter’s and Pell and Gregory classification systems. The distribution of the patients according to episodes, total duration of discomfort, type of impaction, depth of impaction, and Position of Ramus will be evaluated. All the findings will be recorded in a tabular form similar to that shown in Table 1 and Table 2. Statistical analysis will be done using descriptive statistics. The complete analysis dataset will consist of all the participants who have no missing values for any of the parameters.

**Table 1:** Association between mandibular third molar position according to Winter’s classification and recurrent pericoronitis.

Tooth number	Position of the impacted tooth according to Winter’s classification	Number of episodes of pericoronitis	Clinical status of mandibular third molar
1			
2			
3			
4			

**Table 2:** Association between mandibular third molar position according to Pell and Gregory classification and recurrent pericoronitis.

Tooth number	Position of the impacted tooth according to Pell and Gregory classification	Number of episodes of pericoronitis	Clinical status of mandibular third molar
1			
2			
3			
4			

## Discussion

### Principal Findings

This study aims to determine the association between mandibular third molar position and recurrent pericoronitis by using radiographic evaluation. The primary expectation is that certain positional types, as defined by Winter’s and Pell and Gregory classifications, are more frequently associated with recurrent pericoronitis. Identifying these positions may help clinicians predict which partially erupted third molar is at higher risk and requires early intervention.

Pericoronitis is a clinical condition often observed in dental practice. The clinical manifestations range from asymptomatic to quite severe in some cases. Usually, the position or path of eruption of mandibular third molar is responsible for pericoronitis. Therefore, the evaluation of the position of mandibular third molars by radiographic examination is vital for detecting the risk of pericoronitis associated with the erupting or impacted mandibular third molar. Thus, the focus of this study is whether the position of the impacted mandibular third molar by using an orthopantomogram is reliable for diagnosing patients who are at a high risk of developing pericoronitis.

### Comparison With Available Literature

A 2023 study by Shirzadeh et al [2] assessed the prevalence of different types of lower third molar impactions in individuals with pericoronitis in 60 participants. Their findings indicated that class B, class II, and mesioangular impaction types can lead to higher incidence of pericoronitis.

Galvão et al [11] performed a systematic review and meta-analysis in 2019 to determine the association between the lower wisdom tooth position and the presence of pericoronitis. Findings showed stronger correlation between pericoronitis and the vertical position of the lower wisdom tooth compared to other Winter’s classifications. Additionally, within the Pell and Gregory classification, position A had a higher risk of pericoronitis than position B. Their study suggests that the prophylactic extraction of partially erupted lower wisdom tooth, particularly those in position A, is recommended to reduce the risk of pericoronitis.

Prasad [1] concluded that in case of asymptomatic mandibular impacted third molars, the precautionary approach might not be the most effective option. Ongoing monitoring at regular intervals and with the help of patient-centered decision-making, clinicians can develop the most effective treatment plan.

Indira et al [6] reported that the impacted third molar may be associated with the development of pericoronal infectious process and their potential complications and can be advised for prophylactic removal, particularly in position IA, vertical, and distoangular impactions to prevent surgical risks and patients’ morbidity with age.

Singh et al [13] stated that the analysis of the patterns of the impacted mandibular molars may lead to early prediction and diagnosis of the various pathologies associated with the impacted mandibular molar.

Erçal [14] highlighted the significant association between the presence of impacted third molars and various pathological conditions. They also stated the importance of a follow-up to monitor possible changes in the future even though most impacted teeth remained asymptomatic. The third molars were the most prevalent impacted teeth, with approximately 92% of all the impacted teeth, followed by canines and supernumerary teeth. A pathology was detected in 38 out of 335 of the impacted teeth in their study.

Santos et al [15] in their cross-sectional study (2020) evaluated how the position of the mandibular third molars affects periodontal status and clinical severity in patients with acute pericoronitis. They found that teeth in Pell and Gregory position IIA showed greater gingival edema and erythema compared to IA. Increased plaque accumulation, deeper periodontal pockets, and reduced alveolar bone height were observed in adjacent second molars. Their study showed that the third molar position significantly influences both local periodontal health and the clinical severity of pericoronitis.

Nguyen et al [16] analyzed the factors influencing the severity of pericoronitis in patients with impacted mandibular third molars in 2024. Using multivariate analysis, they found that distal radiolucency, right-side involvement in females, upper respiratory infection in males, and menstruation were significantly associated with greater severity. Severity scores correlated with increased pain, swelling, and functional limitation. They concluded that both local radiographic features and systemic conditions contribute to the severity of pericoronitis. Similarly, our study will determine the association between the mandibular third molar position and recurrent pericoronitis. That may help in educating the patient about the condition, thereby aiding in the appropriate treatment approach and halting of the progression of the same.

### Study Limitations

This study will use the orthopantomogram, which is one of the popular commonly used imaging modalities in dental practice; however, the use of cone beam computed tomography will give 3D information regarding the position of the impacted mandibular third molar. The examiner variability in clinical assessments could lead to minor observer bias despite standardization measures. The 12-month follow-up period may also result in participant attrition, affecting data completeness, although adjustments were made during sample size estimation. Finally, this study is institution-based, which may limit the generalizability of its findings to broader populations.

### Broader Implications and Conclusions

Understanding the association between mandibular third molar position and recurrent pericoronitis has important clinical implications. If specific impaction types are found to predispose to recurrence, clinicians can apply radiographic evaluation as a predictive diagnostic tool to identify high-risk patients. This approach can support personalized management strategies such as early prophylactic extraction for high-risk impactions or conservative monitoring for low-risk cases, thereby minimizing patient morbidity and surgical complications. On a broader scale, these findings could guide evidence-based decision-making in oral and maxillofacial practice, reduce unnecessary extractions, and optimize patient outcomes.

### Dissemination of the Study Results

The results of this study will be shared through multiple channels to ensure that all stakeholders, including participants, clinical practitioners, and policymakers, are appropriately informed. Academicians will be informed through presentations at conferences, publications in peer-reviewed journals, and clinical bulletins, facilitating integration of the findings into evidence-based practice. All findings will be presented in a manner that ensures participant confidentiality is fully protected.

## Data Availability

All data generated or analyzed during this study will be made available by the corresponding author upon reasonable request.
